# Epidemiology of 911 Calls for Opioid Overdose in Nogales, Arizona

**DOI:** 10.5811/westjem.18597

**Published:** 2025-03-31

**Authors:** Melody Glenn, Darien Stratton, Keith Primeau, Amber Rice

**Affiliations:** * University of Arizona College of Medicine, Department of Emergency Medicine, Tucson, Arizona; † University of Arizona College of Medicine, Department of Psychiatry, Tucson, Arizona; ‡ University of Pittsburgh, Department of Emergency Medicine, Pittsburgh, Pennsylvania

## Abstract

**Objective:**

Drug overdose is the leading cause of unintentional death in the United States, and individuals identifying as BIPOC (Black, indigenous and people of color) and those of low socioeconomic status are over-represented in this statistic. The US-Mexico border faces several unique challenges when it comes to healthcare and the drug overdose crisis, due in large part to health inequities. Although the US Centers for Disease Control and Prevention recommends that overdose prevention programs address health inequities, little is known about opioid overdoses in this rural, primarily Spanish-speaking region. As emergency medical services (EMS) records collect countywide data, they represent a high-quality source for epidemiologic surveillance.

**Methods:**

We conducted a retrospective chart review based on a local quality assurance program in which two years of EMS records were reviewed with the primary objective of characterizing patients receiving prehospital care for opioid overdoses in a rural, borderland community, and the secondary objective of characterizing EMS’s fidelity to a naloxone distribution protocol. We included electronic patient care records for analysis if they included the EMS clinician’s impression of overdose, opiate abuse, or opiate-related disorder from November 1, 2020–October 31,2022. The following data points were abstracted: date; patient initials/gender/age; police presence; response location; bystanders on scene; naloxone administration prior to EMS arrival; distribution of naloxone kit (yes/no); substance reported; and disposition. We analyzed descriptive statistics.

**Results:**

A total of 74 cases met inclusion criteria over two years with the majority of cases involving men (82%) with a median age of 28. Almost half of overdoses occurred at private residences (46%), and slightly more than half (57%) reported fentanyl use prior to overdose. Family or friends were usually (64%) on scene, and law enforcement was often (77%) the first 911 to arrive. Naloxone was administered on scene in almost all cases (91%), usually by EMS (44%) or law enforcement (43%). The EMS clinicians distributed naloxone kits at 61% of calls.

**Conclusion:**

Opioid overdoses along the US-Mexico border occurred primarily among young men using illicit fentanyl in private residences. Although family/friends were often present, they rarely administered naloxone. Law enforcement was often the first 911 responder to arrive. Emergency medical services is a suitable setting for naloxone distribution programs.

## INTRODUCTION

Although modern emergency medical services (EMS) systems were largely developed to respond to motor vehicle collisions (MVC), opioid overdoses have since surpassed MVCs as the leading cause of accidental death. Yet beyond protocols delineating how to reverse an acute opioid overdose with naloxone, many EMS agencies do not offer prevention or treatment for people who use opioids chaotically. Fortunately, more and more agencies are beginning to initiate buprenorphine and offer a wide spectrum of harm reduction services.

Additionally, EMS is uniquely situated to reach a large population of people who use drugs. In one study of 218 individuals who died from unintentional opioid overdose, 30% had used EMS within the year preceding their death.^1^ Furthermore, EMS may have been these patients’ only point of contact with the healthcare system. One study demonstrated that approximately one third of patients who received prehospital naloxone subsequently refused transport to the emergency department (ED).^2^,^3^ This trend is alarming, as this is a high-risk population with roughly 10% dying in the year following an overdose-reversal by EMS.^16^ Emergency medical services is uniquely positioned to both gather uncaptured epidemiologic data about the opioid epidemic and provide effective prehospital interventions for chaotic opioid use.

One role for EMS in stemming the opioid epidemic includes opioid education and naloxone distribution (OEND), an intervention in which laypeople learn how to recognize an opioid overdose and administer naloxone and are given a naloxone kit. As 95% of people who could benefit from substance use disorder (SUD) treatment (buprenorphine or methadone) do not think they need it, ubiquitous naloxone distribution as a form of harm reduction is paramount.^4^ Additionally, even people in recovery occasionally relapse.

As community nonprofits were the first organizations to implement OEND programs, they are the most studied, showing a reduction in overdose deaths and increased entry into treatment for opioid use disorder (OUD).^5^,^6^ It is likely that these positive outcomes can be extrapolated to healthcare-based OEND programs if clinicians are willing to distribute the naloxone. Unfortunately, one ED OEND study showed that only 30.9% of eligible patient encounters resulted in naloxone provision, suggesting that there may be other barriers to consider in healthcare-based OEND initiatives.^7^

While the feasibility of prehospital naloxone distribution programs in urban communities^8^ has been studied, little is known about unique rural populations such as those in the borderlands, where additional binational, social, and demographic challenges may affect persons with OUD and the EMS services that serve them. Our study setting, Nogales, AZ, is the largest city in Santa Cruz County and shares an international border with the larger city of Nogales, Sonora, Mexico. Nogales, AZ, has a population of almost 20,000 residents, with nearly 95% identifying as Hispanic/Latino, 90% speaking a language other than English at home, and 41.3% born abroad.^9^ The median household income is $31,997, and 29.8% live below the poverty line.

Rural borderland communities face unique health inequities regarding risk for substance use disorder (SUD) and negative health outcomes, and they have less access to treatment. These communities have several of the characteristics listed by the US Centers for Disease Control and Prevention (CDC) as ones disproportionately affected by the drug overdose crisis: reduced economic stability; limited access to healthcare; limited access to SUD treatment; non-English speaking populations; rural density; and racial/ethnic minority groups.^10^ Although the CDC recommends incorporating targeted prevention strategies that address key drivers of health inequities into overdose prevention programs, first we must better understand these communities. As EMS records collect countywide data, they represent a high-quality source for providing epidemiologic surveillance data^11^,^12^ that can be used to create targeted prevention strategies.

**Population Health Research Capsule**

What do we already know about this issue?

*The US-Mexico border faces unique challenges in the opioid epidemic. Although EMS reach a large population that uses drugs, few patients receive naloxone kits.*

What was the research question?

*In the borderlands, what is the epidemiology of patients whose overdose is reversed by EMS? Will EMS clinicians distribute layperson naloxone?*

What was the major finding of the study?

*Family or friends were usually (64%) on scene yet infrequently administered naloxone. Law enforcement was often (77%) the first 911 responder to arrive. EMS clinicians distributed naloxone kits during 61% of their calls for overdose.*

How does this improve population health?

*In rural, borderland communities, we should increase overdose recognition and reversal training to laypeople. Law enforcement involvement should be further studied. EMS is a suitable setting for naloxone distribution programs.*

Our fire department-based EMS agency developed a protocol in 2020 by which patients deemed at risk of opioid overdose could receive EMS-driven education around opioid use, information about addiction treatment facilities in the region, linkage to peer support, and a naloxone kit. As part of a quality assurance program, the medical director reviewed the electronic patient care records (ePCR) for all 911 overdose responses from 2020–2022 to better understand this patient population, identify issues with the protocol, and offer feedback to involved crew.

Our primary objective was to better describe the patients who are cared for by EMS in our rural, borderland community after a suspected opioid overdose to gain additional epidemiologic data that can subsequently be used to improve prehospital harm reduction and addiction treatment initiatives in this unique population. Our secondary objective was to characterize fidelity to the naloxone distribution component of our agency’s prehospital protocol by calculating the percentage of eligible patients who received a take-home naloxone kit.

## METHODS

### Setting

There is one hospital in the city of Nogales and Santa Cruz County, AZ, and at the time of data collection it did not have buprenorphine on formulary or offer naloxone distribution. Similarly, there is only one opioid treatment program (OTP) offering methadone and buprenorphine, and one federally qualified health center that functions as an office-based opioid treatment (OBOT) program that offers buprenorphine for its patients. There are no harm reduction nonprofits based in the county, although a statewide nonprofit provides intermittent outreach. As of 2018, Arizona state regulations authorize law enforcement officers (LEO) to administer naloxone, and intranasal naloxone is carried by some trained LEOs.

At the time of data collection, 911 dispatch was provided by the Santa Cruz County Sheriff’s Office, which did not use medical protocols or pre-arrival instructions such as those used by the Medical Priority Dispatch System. When someone calls 911 for an overdose, law enforcement is often dispatched along with fire/EMS. The Nogales Fire Department (NFD) is the exclusive 911 EMS provider for the city, with an annual call volume of 3,678 in 2021. All firefighters are certified as either an emergency medical technician or a paramedic.

In September 2020, all EMS personnel at the NFD received 2.0 hours of continuing education on OUD, naloxone/harm reduction, medications for OUD, community referrals, and their OEND protocol. The training included virtual and in-person components taught by their EMS medical director who is board-certified in both EMS and addiction medicine, the local OTP, OBOT professionals, and a peer support specialist (PSS). Medical director involvement and in-person elements were designed to convey the clinical value of such an intervention and foster community-wide, interdisciplinary collaboration across the healthcare system.

In October 2020, the OEND protocol was initiated. Those eligible for the OEND protocol (Figure) included any person at risk of an opioid overdose and bystander witnesses of an overdose. The EMS clinicians provided a naloxone kit (intramuscular or intranasal), taught participants how to recognize an overdose and administer naloxone, and explained local treatment options. If EMS clinicians administered naloxone for an acute overdose, they were instructed to call HOPE, Inc, a nonprofit that provides outpatient treatment for mental illness and SUD, for a warm handoff by a PSS. Although the PSS worked traditional office hours, they had a HIPAA-compliant voicemail. To increase the likelihood of a patient being linked to a PSS, the call was an opt-out process. If it were opt in, patients who were unable to have a significant conversation due to sedation or precipitated to withdrawal might be less likely to receive this linkage to care. Even patients transported to an ED were supposed to be linked to PSS, as they provide additional services not found in most EDs.

### Selection of Participants and Outcomes

The prehospital coordinator queried all NFD ePCRs to find patients treated by NFD with a primary or secondary impression from the prehospital clinician of “overdose,” “opiate abuse,” or “opiate related disorder” from November 1, 2020–October 31, 2022 with the objective of identifying all charts related to a non-fatal opioid overdose, as these are the most high-risk patients among those with chaotic opioid use. All included ePCRs were then reviewed by the EMS medical director who placed the following data in a spreadsheet: date of call; patient initials; patient gender; patient age; police presence on scene; location of the overdose/response; type of bystanders on scene (if any); naloxone administration prior to EMS arrival and by whom; whether a naloxone kit was left with the patient; substance used (per patient/bystander report or response to naloxone administration); and patient disposition. Much of this information was obtained from the narrative section of the ePCR.

In some cases, patients denied drug use or declined to state what they had used. In those cases, the spO_2_, respiratory rate, Glasgow Coma Scale, and the ePCR narrative were reviewed by the medical director to look for signs of opioid exposure, such as a clinical response to naloxone (nausea/vomiting or increased GCS, RR, SpO_2_), the presence of drug paraphernalia such as the presence of the foil/lighter used to smoke fentanyl, or history/signs of drug use (such as track marks). Records were excluded if the medical director determined they were not likely related to illicit drug ingestion (for example, an acetaminophen overdose as a suicide attempt would be excluded) or if they were pronounced dead on scene (such as a cardiac arrest). Each week, the medical director sent specific feedback to the involved crew for quality assurance. To evaluate our secondary objective of characterizing the fidelity to the naloxone distribution component of the protocol, we calculated the rate of naloxone distribution among our included ePCRs.

We extracted data from the ePCR as noted above and entered it into an Excel spreadsheet v16.71 (Microsoft Corporation, Redmond, WA). Fentanyl use was classified through subject or EMS clinicians noting use of fentanyl or M30, the street name for illicitly fabricated fentanyl pills in the area. Subject drug use was classified as non-fentanyl opioids if the subject or EMS clinicians noted use of a different opioid or if paraphernalia of IV heroin use were found on scene; and drug use was classified as unknown if subjects denied using opioids or endorsed using non-opioid illicit substances but had a clinical response to naloxone. Location of EMS call was classified as residential; commercial if at a business; public if in a parking lot or at a public facility such as a park; vehicle if called to a parked vehicle; at a healthcare facility if at a community health center such as a pharmacy; or in custody if the subject was in custody of the Nogales Police Department, Department of Corrections, or detained by US Customs and Border Protection.

### Analysis

We performed statistical analysis using Stata statistical software version 16.1 (StataCorp, College Station, TX). Subject characteristics are reported with descriptive statistics presented as medians with measures of dispersion for continuous variables and proportions for categorical data. We compared the incidence of leave-behind naloxone administration among groups using relative risk (RR) and 95% confidence intervals (CI) calculated using the Fisher exact test. The study received human subjects review exemption from the institutional review board of the University of Arizona Human Subjects Protection Program.

## RESULTS

Of 82 records with the target primary/secondary impressions, 74 records met inclusion criteria. The median age of patients was 28 (interquartile range 22–35), and 13 (18%) were female. The majority (57%) reported the use of fentanyl prior to their overdose, 12% endorsed using a non-fentanyl opioid, and 31% used unknown substances or denied substance use (Table 1).

Overdoses most commonly occurred at a private residence (46%) with family members or friends on scene 64% of the time calling 911 (Table 2). Law enforcement officers were frequently the first dispatched responder on scene, representing 77% of study cases. In almost all (91%) of the recorded EMS calls, patients were administered naloxone for suspected overdose reversal. Of those receiving naloxone, 12 (18%) were administered naloxone by multiple parties, whether bystanders, LEOS, or EMS. The median number of naloxone doses was two (range 0–10), with 31% of patients requiring only a single dose. When naloxone was administered, it was administered primarily by EMS (44%) and LEOs (43%), including Nogales Police Department, Department of Corrections, or Customs and Border Protection, and by family/bystanders in 10% of cases. In our study, 19% of patients refused subsequent transport to the ED, which is consistent with refusal rates observed in the literature.^3^ Leave-behind naloxone was provided in 61% of overdose calls, including those in which naloxone was not administered in the field.

Subjects who refused EMS transport to the hospital were 1.74 (RR) times as likely to receive leave-behind naloxone from EMS as those who were transported to the hospital (95% CI 1.32–2.3). Subjects who had family or friends on scene with EMS were 2.29 times as likely to be given leave-behind naloxone as those who did not (95% CI 1.31–3.40).

## DISCUSSION

The patients in our study frequently used at home around family, yet family and friends administered naloxone at relatively low rates compared to professionals who arrived later. This highlights the importance of training family members regarding overdose recognition/reversal and providing them with naloxone to prevent future overdose deaths. Additionally, we found that patients who were not transported to the emergency department following overdose were almost twice as likely to receive a leave-behind naloxone kit, which illustrates the impact that prehospital clinicians may have on patients who do not interact with the healthcare system further. To address these needs, the NFD has become an active member of a countywide substance use consortium and increased its community outreach, with large placards outside the fire stations highlighting that they are Safe Stations; radio advertisements welcoming people to pick up free naloxone; and tabling at overdose prevention events. In positioning itself as a key player in combating the opioid epidemic in the community, NFD aims to reduce the stigma that many persons who use drugs (PWUD) face when accessing healthcare and enhance the trust between them and EMS. This is especially important given the number of patients that decline transport after high-risk overdose events. Further studies could evaluate family and friends’ beliefs and behaviors around layperson naloxone in rural, borderland communities like ours.

Our study also showed that LEOs often arrived on scene before EMS and played a substantial role in overdose recognition and naloxone administration in our community. To better understand the clinical significance of law enforcement’s actions, it would be useful to know just how much sooner they arrived. If it were several minutes, it would suggest that LEOs should receive enhanced training on the medical model of substance use, harm reduction, and overdose recognition/reversal. However, if it were less than a minute, perhaps the automatic dual dispatch to overdose calls should be reconsidered, as one minute may not confer enough clinical benefit to justify the cost of the increased trainings and law enforcement response. Instead, perhaps a community’s financial resources would be better spent on training bystanders.

Additionally, removing law enforcement from overdose calls might reduce existing anxieties of PWUD about calling 911, especially in a region where citizenship and residency concerns are also prevalent.^14^ Although many states have tried to reduce this barrier by implementing Good Samaritan laws that provide limited criminal immunity to people who request help for an overdose, their effectiveness has been mixed.^14^ As their specific provisions vary by state, some offer more protections than others and many PWUD are still hesitant to call 911. This is unfortunate, as EMS can both save a life from a specific overdose event and potentially offer education, harm reduction, treatment, and further linkage to care.^15^

Future initiatives could include expanding the role of EMS in harm reduction for opioid overdoses, such as the distribution of fentanyl and xylazine test strips and safe use supplies, staffing overdose prevention centers, referral to harm reduction specialists, the use of alternate destinations to overdose prevention centers, and expanding the role of EMS in addiction treatment with buprenorphine induction and the use of alternate destinations to opioid treatment programs. Future prehospital addiction research should evaluate what interventions interest prehospital patients (perhaps patients who have suffered a prehospital overdose are more interested in SUD treatment than the 5% identified by the National Survey on Drug Use and Health among the general using population)^5^ and the short- and long-term outcomes of patients who received EMS intervention and/or referral for suspected OUD.

Our findings also suggests that EMS can be a suitable environment for naloxone distribution programs, as 61% of our system’s overdose calls resulted in the provision of naloxone. Compared to the ED-based study^7^ in which only 30.9% of eligible patients received a naloxone kit, this seems like a success. Although we hoped this number would be closer to 100%, there were some situations that made naloxone distribution difficult. For example, for the four patients who experienced an overdose while in the county detention facility/jail, it was not clear whether EMS could leave naloxone with them, and so they did not. Further research could evaluate EMS clinicians perspectives regarding OEND programs; training elements that increase fidelity to OEND protocols; whether EMS-based OEND programs shift EMS clinician opinions about PWUD and overdose calls, and whether fidelity to an OEND protocol decreases as the time from training increases.

## LIMITATIONS

This study has several limitations. This was a descriptive, retrospective study with a small sample size. It is possible that some patients who experienced an opioid overdose were missed due to a different impression documented by the EMS clinician, although due to education provided, this number is likely to be very small. Additionally, not all patients who received the primary/secondary impression of “overdose” actually experienced an overdose; that is, not all had respiratory or central nervous system depression to the extent that they required naloxone. However, all included patients did experience some history or symptoms suggestive of opioid ingestion.

Additionally, there was variability in the manner in which EMS clinicians classified locations and bystanders within the ePCR, as this was not a prospective study with explicit training on classification; much of this information was found within the narrative. Similarly, there may have been bystanders present on scene, but if they were not mentioned in the ePCR narrative, this would not have been captured. Intoxicant drugs were not tested to confirm the underlying substance, and there may be inaccuracies in subject reporting of what was used. Finally, this information may not be generalizable to all rural communities in the borderlands, as Tucson and the University of Arizona are nearby and support several social service programs in the area. Although our department was able to receive free training and naloxone, cost may be more of a barrier for other agencies.

## CONCLUSION

In this rural community along the US-Mexico border, opioid overdoses occurred primarily among young men using illicit fentanyl in private residences. Even though family was often present, they rarely administered naloxone. In our community, law enforcement officers often arrived first on scene and played a substantial role in overdose recognition and reversal. With 61% of eligible patients receiving naloxone kits from EMS, the prehospital setting is well suited for such an opioid education and naloxone distribution protocol.

## Figures and Tables

**Figure:**
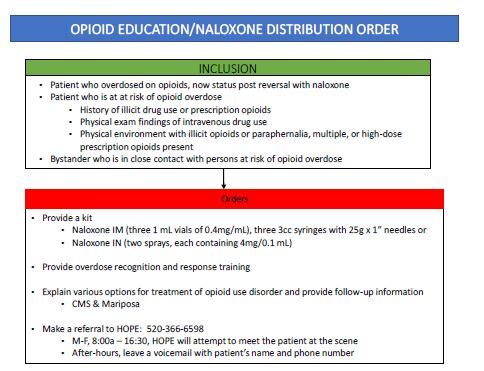
Opioid education and naloxone distribution protocol. *CMS, Community Medical Services; IM*, intramuscular; *IN*, intranasal; *IVDU*, intravenous drug use; *Mariposa*, Mariposa Community Health Center; *s/p*, status post.

**Table 1:** Demographics of emergency medical services calls with primary or secondary impression of opioid overdose in Nogales, Arizona, October 2021–2022.

Variable	No (%)
Age, median (IQR), y	28 (22–35)
Gender	
Female	13 (17.6)
Male	61 (82.4)
Opioid	
Fentanyl	42 (56.8)
Other opioid	9 (12.2)
Unknown	23 (31)
Law enforcement presence on scene	
Yes	57 (77)
No	17 (23)
Naloxone administration	
Yes	67 (90.5)
No	7 (9.5)
EMS disposition	
Hospital transport	60 (81.1)
Transport refusal	14 (18.9)
Naloxone kit distributed	
Yes	45 (60.8)
No	29 (39.2)

*IQR*, interquartile range.

**Table 2:** Scene characteristics and by whom naloxone was administered on scene for emergency medical services calls with primary or secondary impression of opioid overdose in Nogales, AZ, October 2021–2022.

Variable	No (%)
Primary bystander on scene at EMS arrival	
Family/significant other	37 (50)
Law enforcement	15 (20.3)
Friend	10 (13.5)
Employee of business	2 (2.7)
Unknown	6 (8.1)
Healthcare worker	1 (1.3)
None	3 (4.1)
Overdose location	
Private residence	34 (46)
Private vehicle	10 (13.5)
In custody	12 (16.2)
Public	8 (10.8)
Commercial	7 (9.5)
Healthcare facility	3 (4)
Naloxone administration	
EMS	35 (44.3)
NPD	31 (39.2)
Family	8 (10.1)
CBP	1 (1.3)
Department of Corrections	2 (2.5)
Self	1 (1.3)
Healthcare worker	1 (1.3)

*EMS, emergency nedical services; NPD*, Nogales Police Department; *CBP*, Customs and Border Protection.
